# Enzymatic Methods for Salivary Biomarkers Detection: Overview and Current Challenges

**DOI:** 10.3390/molecules26227026

**Published:** 2021-11-20

**Authors:** Alonso Ornelas-González, Margarita Ortiz-Martínez, Mirna González-González, Marco Rito-Palomares

**Affiliations:** Escuela de Medicina y Ciencias de la Salud, Tecnologico de Monterrey, Av. Morones Prieto 3000, Monterrey 64710, N.L., Mexico; A00821703@itesm.mx (A.O.-G.); margarita.ortiz.mtz@tec.mx (M.O.-M.)

**Keywords:** enzyme assays, saliva, biomarkers, analysis

## Abstract

Early detection is a key factor in patient fate. Currently, multiple biomolecules have been recognized as biomarkers. Nevertheless, their identification is only the starting line on the way to their implementation in disease diagnosis. Although blood is the biofluid par excellence for the quantification of biomarkers, its extraction is uncomfortable and painful for many patients. In this sense, there is a gap in which saliva emerges as a non-invasive and valuable source of information, as it contains many of the biomarkers found in blood. Recent technological advances have made it possible to detect and quantify biomarkers in saliva samples. However, there are opportunity areas in terms of cost and complexity, which could be solved using simpler methodologies such as those based on enzymes. Many reviews have focused on presenting the state-of-the-art in identifying biomarkers in saliva samples. However, just a few of them provide critical analysis of technical elements for biomarker quantification in enzymatic methods for large-scale clinical applications. Thus, this review proposes enzymatic assays as a cost-effective alternative to overcome the limitations of current methods for the quantification of biomarkers in saliva, highlighting the technical and operational considerations necessary for sampling, method development, optimization, and validation.

## 1. Biomarkers as a Diagnostic Tool

A biomarker, also known as a biological marker, is any molecule, substance, or measurable process in the body or in one of its components that can support the diagnosis, prognosis, prediction, or response to the treatment of a disease [1]. Unlike symptoms, which often are endpoints of the disease, biomarkers appear, disappear, or vary in concentration from the onset of the disease, so they are useful for detection at earlier stage [2]. The use of biomarkers increases the reliability of the diagnosis, helping to provide more effective and safer treatments for patients. A “good” biomarker must be present in easily collectable samples, which can be processed and stored without affecting its concentration. Furthermore, its concentration must be easily measured and constant (ideally with low variability) in the control population (healthy) and altered in the diseased population [3].

Multiple steps are required to develop a biomarker quantification method, but biomarker identification is the starting point for its clinical application. Likewise, the method has to be standardized, optimized, and validated before its large-scale implementation as a diagnostic tool. In this context, advances in metabolomics and analytical techniques have been exploited, resulting in the discovery of hundreds of thousands of metabolites [4]. Different techniques such as high-performance liquid chromatography (HPLC), gas chromatography (GC), nuclear magnetic resonance (NMR), and mass spectrometry (MS) are used for the identification and quantification of biomarkers. 

**Figure 1:**
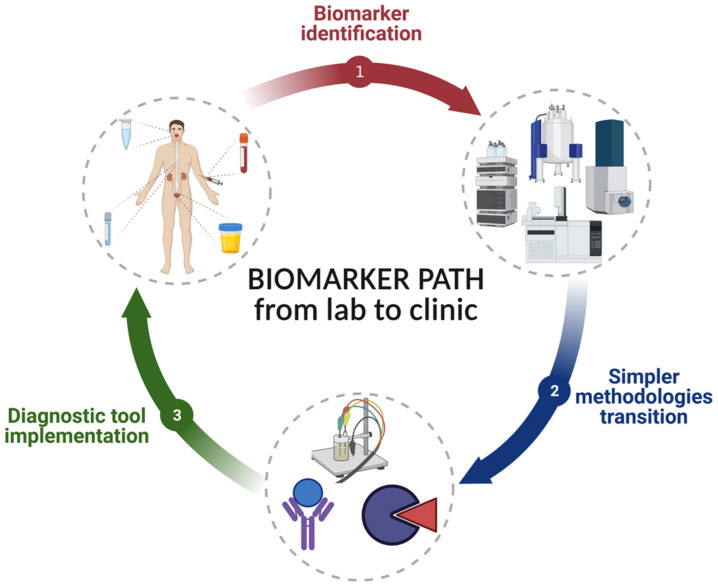
Schematic representation of the biomarker pathway from the laboratory to the clinical applications. (1) Collection of biological samples to identify and quantify biomarkers using sensitive techniques such as mass spectrometry, gas and liquid chromatography, and nuclear magnetic resonance. (2) Transition from the already established methodologies to more straightforward and inexpensive methods based on antibodies, enzymes, and electrochemistry. (3) Validation and implementation of these methodologies as a diagnosis tool.

Following the method development pathway, the identified biomarkers must be correlated with a disease [5]. For this, a very broad knowledge of both the pathophysiology of the disease and the origin of the signaling molecule is necessary [6]. As biomarkers are signaling molecules present in different biofluids in varying concentrations, the selection of a biofluid among the more than 30 found in the body [7] depends on several factors, such as chemical nature, stability, and the concentration of the biomarker in the biofluid, as well as its ability to reflect the presence, persistence, and evolution of the disease. Moreover, it should be considered that its success also depends on the degree of acceptance by the patient, leading to widespread use [8]. Therefore, this review aims to show a global vision of the biomarker pathway from the laboratory to the clinical applications (Figure 1), proposing enzymatic assays as a cost-effective alternative to overcome the limitations of current methods for the quantification of biomarkers in saliva, highlighting the considerations techniques and operations necessary for sampling, method development, optimization, and validation. 

## 2. Advantages of Saliva Samples as a Biomarker Source

Early detection is a key factor in patient fate. Blood is undoubtedly one of the most used biofluids for measuring biomarkers [9]. Its function as a carrier of cells, gases, nutrients, biomolecules, and waste through the body becomes a valuable source of information regarding the health condition of a patient [10]. However, its extraction implicates an invasive and painful procedure that requires trained personnel. Likewise, this practice generates biological waste, as special supplies such as gauze pads, syringes, and tubes are required. In this regard, other biofluids such as saliva, urine, and sweat have gained ground in the search to minimize possible nonconformities for the patient and simplify the collection method of the biofluid.

Similar to blood, saliva acts as a mirror that reflects the physiological state of the organism. Saliva is an intercellular ultrafiltrate from the blood [11]. It is mainly constituted by water (99%); however, it is an enormously complex fluid, containing more than 850 non-redundant metabolites [12], including proteins, electrolytes, mRNA, DNA, enzymes, antibodies, sugars, hormones, and other molecules [13]. It is known that salivary compounds can drastically change their concentration in consequence of a great variety of physiological states, stimuli, and stress states, so their identification and quantification can be useful for their early diagnosis [14]. Moreover, as its extraction does not require privacy as urine does, it is not difficult to stimulate as tears are, and it does not involve physical effort as sweat does; saliva has gained acceptance and stands out over other biofluids [10,15]. 

In general terms, saliva samples can be collected under an uncomplicated procedure with minimal risk of cross-contamination (if taken under the right conditions). In addition, it has the advantage of requiring less preparation for analysis and less space for storage than blood samples [16]. All these advantages are remarkable and postulate saliva as an attractive source of information for the early quantification of biomarkers in patients with little tolerance to blood collection [17], and this is perhaps one of the reasons for its rise as an alternative biofluid to blood. There are different techniques for saliva sampling, such as spitting, collecting it with the help of a sponge or other device, or directly from the salivary gland duct [18]. The choice of the sampling procedure relies on several factors such as the biomarker type, the quantification method, the equipment availability, and other operational and economic aspects. 

While saliva appears to be a valuable diagnostic tool, there are some methodological concerns that must be taken into consideration. For instance, the widespread use of techniques such as RNA sequencing (RNA-Seq) for the profiling of RNA-based biomarkers opens a new window in the search for useful targets for the diagnosis of diseases. However, this implies facing new challenges due to the high abundance of bacterial content and low abundance of salivary RNA [19]. The next section of this review article addresses the identification process and initial quantification of biomarkers in saliva, highlighting the bottlenecks in the most widely used techniques to identify a route to generate cost-effective diagnostic methods. 

## 3. Current Methods for Salivary Biomarker Identification

The identification and development of preliminary profiles is the first step in the discovery of biomarkers. These stages are generally accomplished by coupling powerful analytical techniques such as GC-MS and liquid chromatography-MS (LC-MS) [7]. This section of the article highlights the strengths of the most widely used analytical techniques for the identification and profiling of salivary biomarkers, as well as methodological aspects that preclude their implementation as a large-scale diagnostic method.

The techniques used for the identification and biomarkers profiling vary in terms of operating principle, sample preparation, and results interpretation, on which the suitability of its implementation depends [20]. For instance, MS can measure hundreds to thousands of metabolites in widely varied samples such as tissues, blood, urine, cerebrospinal fluid, and saliva, being suitable for untargeted and targeted biomarker screening and profiling [21]. However, it should be considered that this technique ionizes the sample in order to identify its components, so it could not be used for further studies if necessary.

Another technique commonly used in metabolomics for biomarkers discovery in saliva samples is NMR. This robust and reproducible technique has a series of advantages over others. For instance, it can detect highly volatile metabolites and does not require derivatization of the compound to increase its detectability as in GC [22]. Despite that sample preparation is less labor-intensive than LC, its sensitivity is lower than coupled techniques such as GC-MS and LC-MS, which can detect biomarkers below the detection limit of NMR [23]. Furthermore, it should be considered that saliva is a complex matrix, so previous pre-treatment steps of filtration and/or centrifugation are required.

Capillary electrophoresis-MS (CE-MS) is an alternative technique for biomarker identification in saliva that has recently grown in acceptance [24]. This method fusions the ability of electrophoresis to separate compounds by their electrophoretic mobility in the function of an applied voltage with the sensitivity of MS, resulting in an attractive and powerful system [25]. Thus, its use has increased rapidly, resulting in more than 50 published articles for metabolite profiling from 2018 to 2020 [26]. This is undoubtedly a reflection of its potential in the field of metabolomics. However, it should be noted that for this technique to be reproducible, multiple problems must be solved to control the loss of metabolites by adsorption processes, volume adjustments, and dilution of the sample [26].

As mentioned, advances in analytical techniques, statistics, and data analysis have resulted in a “boom” in biomarkers discovery. All these techniques show characteristics that make them ideal for quantifying a large number of biomarkers in different samples. However, it should be noted that all are highly sophisticated, requiring highly qualified personnel and perfectly adapted environments for sample analysis. In addition, they require expensive and sensitive equipment that represents a considerable economic investment that is not available for all laboratories. In this sense, its use on a large scale, as in diagnosing highly recurrent diseases, is almost impossible, so simpler and inexpensive methodologies are necessary.

Despite the fact that the aforementioned techniques have been used to quantify several metabolites in saliva, simpler techniques such as those based on antibodies, enzymes, or electrochemistry are required to spread their use in the screening, diagnosis, follow-up, and control of highly recurrent diseases. Among these techniques, enzyme-based ones are of particular interest to this work due to their versatility and low cost. Therefore, in the following sections of this review, the main characteristics and elements that should be considered in the development of these methodologies will be discussed.

## 4. Challenges in Enzymatic Methods for Salivary Biomarkers Detection

The enzymatic activity can accelerate chemical reactions, consuming substrates and generating other compounds [27]. In this sense, all enzymatic assays are based on quantifying the consumption of a substrate or the production of a by-product in a given period of time [28]. Currently, a large number of enzymatic methods for the quantification of biomarkers have been developed [29,30,31]. The success of these platforms lies mainly in their relatively low cost, flexibility, and ability to be implemented for the simultaneous quantification of several biomarkers [32]. Nonetheless, it must be considered that saliva is a complex mixture of compounds so that multiple factors can interfere with the measurement procedure.

**Figure 2:**
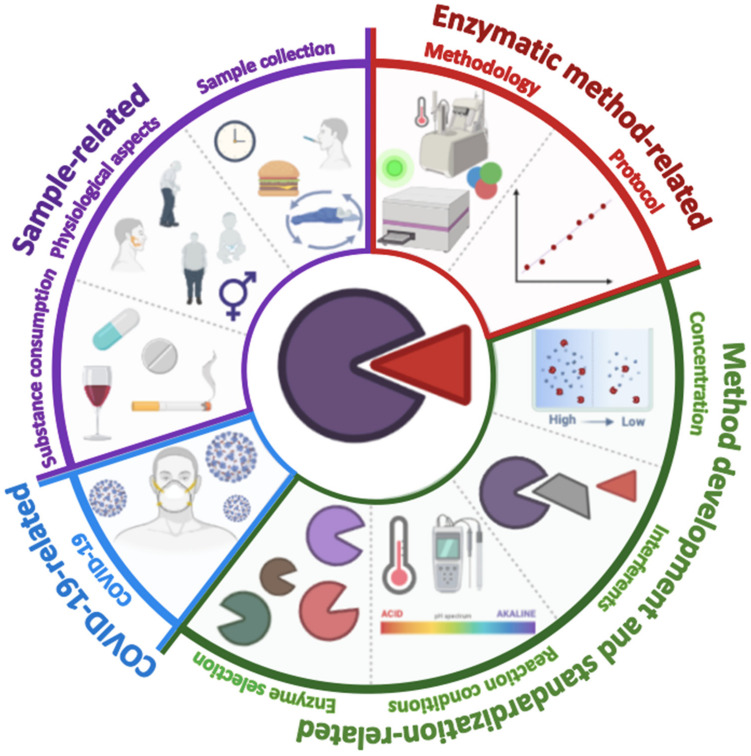
Graphic representation of the main challenges to consider in developing enzymatic methodologies for quantifying salivary biomarkers. Sample-related challenges (diet, circadian cycle, sample collection time, sex, age, physical composition, and other anatomical factors such as the size of the salivary glands); enzymatic method-related (type of assay, correlation degree, and limits of detection and quantification); extrinsic factors (the type of enzyme, the concentrations of enzyme, substrate and other reagents, and the presence of interferents); COVID-19 related factors (precautions and security measures in the collection, processing, and disposal of the sample).

During the following sections of this review, challenges related to the sample, type of enzymatic assay, and method standardization, which play a leading role in the enzymatic determination of biomarkers in saliva, will be described. Likewise, emphasis will be placed on the biosafety measures that must be implemented due to the current situation of the pandemic caused by the SARS-CoV-2 virus for collecting, processing, and correct disposal of saliva samples. Figure 2 shows a graphical representation of all the challenges and considerations in the development of enzymatic methodologies.

### 4.1. Sample-Related Challenges

In saliva, as in other biofluids, sampling is the initial stage of the analysis process. Unlike blood, where sampling involves special equipment and trained personnel, in saliva, the donor can perform this step through a simpler process [33]. However, it is important to mention that the patient must receive detailed information about the sampling protocol, including the importance of the exact moment of sampling, excluding tooth brushing before collection, and avoiding the ingestion of beverages, food, or any other product such as chewing gum for at least 30 min before collection [13]. Likewise, the person in charge of supervising the collection must reject samples contaminated with blood, which could significantly influence the determination.

On the other hand, several factors largely depend on the biomarker to be quantified and must be identified during the planning and development stages of the protocol to avoid variations. For example, it is well known that some non-controllable factors such as circadian cycle, circa-annual cycle, age, gender, body weight, and size of the salivary glands affect the concentration of metabolites in saliva [34,35]. Although these factors cannot be controlled at the sampling time, information about them can be recorded to explain variations in the results. Other factors such as the hydration level, food intake, medications, visual stimulation, and exercise can be “controlled” by giving instructions to the patient prior to sampling [34,35]. In fact, it is common to provide indications such as avoiding food intake, overhydration, and vigorous exercise 2 h before sampling [36]. Similarly, it is recommended to avoid the use of lipstick, lip balm, or any lip product to avoid interference [37].

There are other factors such as tobacco, alcohol, and the presence of diseases that can modify the composition, viscosity, and pH of saliva, generating discrepancies in biomarker measurements in enzymatic methods [38,39,40]. Alcohol and tobacco are well known for altering the flow of saliva and the concentration of proteins, generating systemic changes that indirectly alter the constitution of saliva [41,42]. In addition to these effects, a study conducted by Dukić et al. (2013) found that alcohol has a very significant effect on salivary pH, reducing it by up to 1 unit in alcoholics [43]. 

Although these factors may seem trivial, they could significantly affect enzyme activity during enzymatic assays and, therefore, the quantification of biomarkers. For example, a study showed evidence that people dependent on tobacco and alcohol have a decrease in alcohol dehydrogenase activity, caused by a synergistic toxic effect on the salivary glands derived from the consumption of these substances [44]. Another research work demonstrated that alcohol consumption can decrease the enzymatic activity of salivary amylase up to 25% [45]. More recently, it was suggested that substances contained in cigarette smoke can destroy macromolecules such as proteins and enzymes, decreasing the self-protective capacity of saliva and making it an easy target for bacterial infections [46]. These observed effects could be intrinsically extrapolated to other enzymes, suggesting a potential source of variation in assays that highly depend on enzymatic activity. In this sense, tobacco can also alter the volume, viscosity, and pH of saliva. This was revealed in a comparative study between smokers and non-smokers, finding that the salivary volume and pH of smokers was lower in non-smokers and the salivary viscosity of smokers was greater in non-smokers [47].

On the other hand, the salivary composition can also be altered under certain pathologies, such as diabetes [48,49], oral infections [50], Sjogren’s syndrome [51], kidney disease [52], cancer [53], and virus infections [54]. The presence of these diseases can contribute to variations in the concentration of salivary biomarkers. For example, one study showed a marked decrease in salivary amylase levels and increased glucose concentration in diabetic patients compared to healthy patients [55]. In addition to the obvious effect of decreased biomarker concentration, these diseases can alter salivary pH, an essential parameter in enzymatic methods. In a study carried out by Seethalakshmi et al., diabetes was directly correlated with the pH value, observing a decrease of more than one pH unit in diabetic patients compared to the control group [48]. These findings can be explained as, during diabetes, an increase in the concentration of sugars in the saliva is observed, which in turn increases the presence of bacteria responsible for cavities and increases oral infections. Similarly, during infectious processes, bacteria can use the sugars, acidifying the medium. This decrease in pH can generate variations in enzymatic determinations if it is not properly studied and controlled.

### 4.2. Enzymatic Method-Related Challenges

Enzymatic methods are usually based on detecting substrate consumption or its generation over a period of time [28]. There are different classifications for enzyme-based methodologies [56]. For instance, they can be classified depending on how enzymatic reactions are studied (initial speed, curve progress, kinetics, among others) or according to how the product is quantified (continuously or discontinuously). Within the entire range of options, particular interest has been placed in continuous methods based on spectrophotometry [57]. These tests quantify the light absorbed by a sample when a beam of light passes through it [58]. In enzymatic assays, the absorbed light changes due to the generation of by-products resulting from enzyme activity in either single or multiple reactions. The simplicity of these methods in terms of material, equipment, and training has contributed to their widespread acceptance and implementation. However, these methods are often inadequate for detecting biomarkers at very low concentrations and other strategies, such as derivatization, are required, complexing the process [59]. 

Fortuitously, other methods, such as those based on fluorescence or chemiluminescence, can overcome the limitations mentioned above by being much more sensitive than the previous ones [60,61]. These methods use different molecules capable of absorbing light and emitting it at a specific wavelength, either by themselves or acquiring this property due to a chemical reaction. Due to this, they are much more sensitive than spectrophotometric tests, but it should be considered that these methods are more expensive as they require equipment with special characteristics, are susceptible to interference by impurities in saliva, and are highly unstable when exposed to light. Table 1 shows examples of the application of these spectrophotometric methods to quantify biomarkers in different biological matrices.

There are other more sophisticated techniques, such as microscale thermophoresis, which combine the precision of fluorometry with the sensitivity and versatility of thermophoresis, resulting in a fast, robust, and flexible platform [72]. Among its characteristics, it stands out that it requires sample volumes of less than 10 µL and can analyze multiple substrates simultaneously [73]. Although the advantages of these tests are remarkable, their implementation is limited by high equipment and operational costs, making it difficult to implement them on a large scale and in poorly equipped laboratories.

Despite the fact that the options are vast, the selection of the method depends on multiple factors that must be previously analyzed by the researchers. For example, if an affordable method for the detection of abundant biomarkers with relative accuracy is required, a colorimetric assay could be a suitable option. On the other hand, if the biomarker is present in lower concentration and high precision is required, the most viable options would be to use fluorescent, luminescent, or microscale thermophoresis methods.

### 4.3. Method Development and Standardization-Related Challenges

As the star players of the game, enzymes are the first factor to consider in the development of enzyme assays. The enzyme selection obviously depends on the biomarker to be quantified; however, this choice involves other important details. It is widely known that enzymes display their maximum activity under certain environmental conditions, and disturbances in these states significantly affect their activity [74]. This atmosphere includes factors such as pH, temperature, ionic strength, concentrations of substrate and enzymes, and interferents. All these parameters have a significant impact on the correct performance of these biomolecules and must be carefully analyzed. This section of the review highlights the effects of these parameters in a brief approach; however, a more comprehensive review of these factors can be found in Bisswanger’s review [75].

Multiple enzymes (especially mammalian-derived ones) have an optimum pH, temperature, and ionic strength close to the physiological conditions (pH of 7.4, temperature of 37 °C, and ionic strength of 0.15 M) [76,77,78]. In saliva, the normal pH ranges from 6.2 to 7.6 (average pH of 6.7) [38]; these pH values are below blood values, and their effect on enzymes should be studied. This phenomenon has been widely documented. For example, a study carried out by Bollella et al. proved the importance of controlling the pH in an enzymatic study in which the activity of fructose oxidase was determined. While at a pH of 5.5, this enzyme showed its maximum activity; an increase in pH towards values higher than 6 caused a drastic decrease in its activity [79]. Despite the fact that many other works in this regard are reported [80,81,82,83], the key point is to emphasize the importance of adjusting this often-undervalued factor. Although buffer solutions are usually sufficient to counteract this effect if two or more enzymes with different optimal pH participate in the assay, this becomes more challenging, and special care must be taken.

Similar to the pH, the temperature is a parameter that has a known influence on enzyme activity. As in any chemical reaction, the rate of the enzyme-mediated reaction is strongly influenced by temperature, (generally increasing the reaction as the temperature increases). However, higher temperatures (over about 55 °C) lead to the denaturation of enzymes and the loss of their activity. It is important to mention that this depends closely on the enzyme selected, as some enzymes have a higher optimum temperature, such as polymerase and helicase (optimum temperature of 70–80 °C) [84]. A study suggests that the loss of activity when the temperature is increased precedes denaturation due to changes in flexibility in the active site [85]. In any case, this parameter should be strictly controlled to avoid intra-experiment variations.

Ionic strength is another factor that must be closely considered in enzyme-based assays. This characteristic is given as a function of the concentration of all the ions present in the solution [86]. In saliva, the ionic strength is crucial as it is strongly related to its viscosity; as the ionic strength of the saliva increases, the viscosity decreases [87]. On the other hand, its influence on enzymes has also been shown in various studies in which a decrease in enzymatic activity was observed, probably due to hindering the movement of the molecules in the medium, slowing down the reaction [76]. Although its effect is significant, its effect can be controlled by conducting conductivity studies.

In enzymatic assays, the balance between substrate concentrations, enzymes, and interferents occupies a central place. This is perhaps one of the main challenges that must be faced when using saliva as a sample as the concentrations of biomarkers are usually low, and it contains multiple compounds that can act as interferers [12]. In general terms, interfering molecules make the process difficult and contribute to variations in the results. This is one of the bottlenecks in using saliva as a biomarker source for disease diagnosis, and it certainly depends on the specificity and sensitivity of the method used. Fortunately, very simple methods such as filtration and centrifugation have shown to greatly diminish the effects caused by some interferents such as food fragments, cell debris, mucus, and turbidity, making it suitable as a diagnostic fluid [88]. For instance, centrifugation effectively diminishes viscosity, helping to significantly separate solids, ensuing in a clear supernatant that is easy to pipet [89].

Many other factors are not discussed in this review. However, they must be meticulously considered in enzymatic assays, such as the correct handling of the sample and methodological aspects related to sample preparation, choice of blanks, incubation and reading times, as well as data processing. All these elements taken together can lead to discrepancies in the intra-day and intra-experiment results, thus the importance of establishing a simple, replicable, and robust protocol.

### 4.4. Challenges in the COVID-19 Era

The emergence of the COVID-19 pandemic has highlighted the importance of efficient and safe sampling strategies for disease diagnosis. In this sense, saliva has been positioned as a valuable tool for this purpose as it is an easily collectible fluid in which several biomarkers can be found. During this review work, various saliva-related challenges have been mentioned and addressed; nevertheless, the challenges related to COVID-19 are undoubtedly new and unexpected.

Despite the fact that the saliva sampling techniques are not new, the current situation has made it necessary to implement additional safety measures to guarantee the protection of both the sampling staff and the donor. Some techniques, such as those based on nasopharyngeal/oropharyngeal swabs, are commonly used for saliva sampling. However, it should be noted that they present a series of limitations regarding the safety of the person who collects it. Due to this, other techniques, such as those based on self-collection, would avoid the risks of contagion and the spread of the disease [90]. As saliva samples can be obtained easily, the apparent solution is to instruct the patient to spit into a sterile vial, minimizing staff involvement in the sampling. After sample collection, participants should be provided with disinfectant wipes and instructed to sanitize the exterior surfaces of the collection tube or device. It is also advisable to implement measures such as separating the participant in an isolated area to avoid personnel exposure.

Along with the collection of the sample, its processing involves risks to staff who must be properly trained for handling the sample and protected with the appropriate security measures such as disposable gloves, gowns, and masks, as well as goggles or face shields [91]. Likewise, disposing of one-time use personal protective equipment as medical waste is essential. Additionally, it is important to treat all samples as potentially infective and dispose of them following the guidelines established for the management of medical waste related to COVID-19 [92,93]. In summary, these are just some of the basic protection recommendations to minimize contamination risks during sampling and sample processing.

## 5. Current Trends and Future Perspectives in Enzymatic Methods for Salivary Biomarkers Detection 

Once the enzymatic methodology has been developed and validated, one of the approaches that allow their utilization in the clinic is to turn it into an easy-to-implement test or device. This has been achieved through collaboration between various disciplines such as microfluidics, biotechnology, nanotechnology, computer technology, signal processing, and microelectronics [94]. The combination of this knowledge has resulted in the emergence of countless portable tests and devices, including biosensors [95,96,97], and even microchips [98]. These developments in the clinical area are beneficial as they can provide diagnostic information in an effective and simple way. In addition, they do not require personnel with professional training, allowing the same patient to carry out their implementation [99].

Portable devices and tests for the non-invasive diagnosis of diseases are a growing focus area in the clinical field. Compared to conventional laboratory tests, they stand out for their simplicity and effectiveness in obtaining results in short periods of time at a low cost [100]. These characteristics make them an ideal alternative for remote or hard-to-reach places where setting up a conventional laboratory is not an affordable option [101]. Furthermore, as they are transportable and do not require high energy consumption, they can be easily transported and used at movable diagnostic points [30,102,103]. 

In saliva, these approaches are very widespread, being used for the diagnosis of several health conditions, including cardiovascular diseases [104], Alzheimer’s disease [105], diabetes [106], oral diseases [107,108,109], and cancer [110]. The technology behind the design and operation of these developments is highly varied. For instance, several attempts propose the use of antibodies to improve the specificity of the test being able to identify quantities in the pM range [111,112,113,114]. Nevertheless, it should be considered that their inclusion increases the test cost, which could limit its widespread use. 

Other approaches, such as biosensors, use an optical, electrochemical, and piezoelectric transducer to convert the chemical signal into an electrical one, which can be directly or indirectly related to the biomarker concentration through a software [115]. These systems have been shown to be effective in detecting biomarkers in saliva in short periods of time and with high accuracy. For instance, a prototype developed in 2010 by Yamaguchi and collaborators allows the precise measurement of salivary cortisol in concentrations between 1–10 ng mL^−1^ in just 25 min [115]. Other studies further integrate these biosensors into medical devices such as mouthguards for constant biomarker monitoring. An example of this type of prototype was reported by Kim et al., who developed a mouthguard that allows the measurement of uric acid levels in saliva in the physiological ranges both in healthy and hyperuricemic patients [116].

Future trends in this area seem to point to improving already functional devices taking advantage of nanotechnology. In this sense, various materials developed using this technology have been tested, looking to increase the specificity and sensitivity of the devices [117]. For example, the inclusion of graphene in these developments could help improve signal transduction due to its extraordinary mechanical and electrical properties [118]. Similarly, gold nanoparticles have become popular as reporter molecules due to their optoelectronic properties, low toxicity, and large surface-to-volume ratio [119]. Other nanomaterials, such as semiconductor quantum dots [120], polymer nanoparticles [121], carbon nanotubes [122], and nanodiamonds [123], have been proposed to improve the functioning of biosensors and microdevices [118,124].

On the other hand, the possibility of using existing technology such as smartphones with high-resolution cameras capable of processing information provides a valuable tool that has not been fully exploited and undoubtedly has a huge potential [125]. Similarly, the current transition to industry 4.0 (I4.0) seems to indicate that the use of IoT (Internet of Things) and Big Data technologies will allow machines to work in connection with one another, and processes can be automated in ways never before seen [126].

Conclusively, it should be mentioned that the future of these approaches in clinical diagnosis largely depends on their development being focused on the needs and acceptance of both medical personnel and patients. In addition, it must be considered that governments and regulatory entities play an essential role in the fate of these products. Finally, in the technical aspect, these developments must meet the scientific and economic requirements that allow their widespread use to be a suitable alternative as a diagnostic tool.

## Figures and Tables

**Table 1:** Examples of the use of spectrophotometric methods for the quantification of biomarkers in biofluids.

Method	Principle	Detection Range	Biofluid	Biomarker	Detection Limit	Reference
Colorimetric	Absorption of radiation in the visible area by colored substances	M–nM	Blood	Glucose	31 µg mL^−1^	[62]
			Saliva	Glucose	0.36 µg mL^−1^	[63]
			Sweat	Cortisol	97 ng mL^−1^	[64]
			Urine	Tyrosine	2.54 µM	[65]
Luminescent	Light emitted by a molecule when receiving radiant energy	mM–nM	Blood	Glucose	80 nM	[66]
			Saliva	Glucose	0.63 nM	[67]
			Urine	Melamine	3.5 ng mL^−1^	[68]
Fluorescent	Light emitted by a molecule when receiving radiant energy	mM–nM	Blood	Glucose	3.7 µM	[69]
			Sweat	Chloride	3 mM	[70]
			Urine	Iodide	100 nM	[71]
